# Dynamic DNA methylation in the brain: a new epigenetic mark for experience-dependent plasticity

**DOI:** 10.3389/fncel.2015.00331

**Published:** 2015-08-25

**Authors:** Paola Tognini, Debora Napoli, Tommaso Pizzorusso

**Affiliations:** ^1^Department of Biological Chemistry, University of California, IrvineIrvine, CA, USA; ^2^BioSNS laboratory, Scuola Normale Superiore di PisaPisa, Italy; ^3^Institute of Neuroscience CNRPisa, Italy; ^4^Department of Neuroscience, Psychology, Drug Research and Child Health Neurofarba, University of FlorenceFlorence, Italy

**Keywords:** epigenetics, gene transcription, ocular dominance plasticity, cytosine hydroxylmethylation, neurodevelopmental disorders

## Abstract

Experience-dependent plasticity is the ability of brain circuits to undergo molecular, structural and functional changes as a function of neural activity. Neural activity continuously shapes our brain during all the stages of our life, from infancy through adulthood and beyond. Epigenetic modifications of histone proteins and DNA seem to be a leading molecular mechanism to modulate the transcriptional changes underlying the fine-tuning of synaptic connections and circuitry rewiring during activity-dependent plasticity. The recent discovery that cytosine methylation is an epigenetic mark particularly dynamic in brain cells has strongly increased the interest of neuroscientists in understanding the role of covalent modifications of DNA in activity-induced remodeling of neuronal circuits. Here, we provide an overview of the role of DNA methylation and hydroxylmethylation in brain plasticity both during adulthood, with emphasis on learning and memory related processes, and during postnatal development, focusing specifically on experience-dependent plasticity in the visual cortex.

## Introduction

Phenotype is the result of the dynamic and continuous interaction between genes and environment. Our brain is an excellent system to assess how changes in the external stimuli can influence the structures and the function of a complex organ. Neuronal circuits are able to dynamically refine in response to the huge amount of signals that we are exposed to from our birth (and before) through the rest of our life, resulting in the complexity of our behavior.

The new field of epigenetics seems to be perfect to explain the molecular mechanisms underlying one of the most exciting properties of the brain: its plasticity. Experience-dependent plasticity is the capacity of neuronal circuits to remodel themselves and undergo modifications based on changes in activity and sensory inputs. Plastic phenomena are present at different levels: structural, such as changes in the circuitry and in synaptic connections, or molecular such as modifications in the chromatin landscape and ultimately in gene expression. Brain cells depend on complex and highly regulated mechanisms to appropriately activate or silence gene programs in response to inputs from the environment. These events are controlled by activity-dependent signaling pathways that mediate gene expression by modifying the activity, localization, and/or expression of transcriptional-regulatory enzymes in combination with alterations in chromatin structure in the nucleus (McClung and Nestler, 2008). Therefore, epigenetic mechanisms seem to play a key role in sustaining the transcriptional program responsible for the establishment and refinement of synaptic connections and neuronal circuits especially during development but not only. Recent studies indicate that alterations in chromatin state and gene expression are important for mediating various aspects of experience-dependent plasticity, such as developmental plasticity (e.g., visual cortical plasticity), learning and memory, and maladaptive plasticity (e.g., drug of abuse responses).

Conrad H. Waddington (1905–1975) was the first to introduce the idea of an “epigenetic landscape” (Waddington, 1957), to represent the process of cellular decision making during development (Goldberg et al., 2007). The modern idea of “epigenetics” goes beyond this concept and is defined as the study of heritable changes in gene expression that occur without changes in DNA sequence (Wolffe and Guschin, 2000). Nevertheless the concept of an “epigenetic landscape” might be applied to differentiated cells like neurons that are continuously targeted by different inputs and choose their fate (to make or not a synaptic contact, to spike or not, to wire in a particular circuit) in accordance to the predominance of specific environmental stimuli that ultimately remodel the chromatin structure to activate or silence gene expression.

Neuroepigenetics has been a growing field for the last decade and its research is converging on the study of covalent and noncovalent modifications of DNA and histone proteins and the mechanisms by which such modifications influence overall chromatin structure, gene programs, neuronal differentiation and plasticity, synaptogenesis and finally complex behaviors. Due to important discoveries in the last few years, in our review, we are focusing on covalent DNA modifications especially methylation and hydroxylmethylation involved in experience-dependent brain plasticity.

## DNA Methylation and its Regulation

DNA methylation is a covalent modification that occurs on cytosine mostly located in CG dinucleotides (CpG) by means of a reaction catalysed by a family of enzymes called DNA methyltransferases (DNMT). This modification is known to have a role in the constitutive silencing of chromatin regions, the inactivation of one of the X chromosomes in females, the imprinting of parental alleles, and the silencing of retroviral genes and other individual genes (Ng and Bird, 1999). DNMT, a highly conserved family of proteins, transfer a methyl group from S-adenyl methionine (SAM) to the fifth carbon of a cytosine residue to form 5-methyl-cytosine (5mC; Moore et al., 2013). In mammals, there are three major DNMT: DNMT1, DNMT3a and DNMT3b. DNMT3a and DNMT3b, the so-called *de novo* DNMT, were cloned by Okano et al. (1999) and are responsible for establishing the pattern of methylation in embryonic development. These two enzymes show equal activity toward both unmethylated and hemimethylated DNA (Okano et al., 1999). On the other hand, the maintenance enzyme, DNMT1, shows a strong preference for hemimethylated sites, generated during DNA synthesis (Hermann et al., 2004). DNA methylation and its enzymes have been extensively investigated in the development of the embryo. Intriguingly, postmitotic neurons express high levels of DNMT suggesting a different and new role for these proteins and 5mC in the brain (Goto et al., 1994; Feng et al., 2005).

DNA methylation has been thought to be a static epigenetic mark for over 20 years. Recent evidence demonstrated that is dynamically regulated both through passive and active mechanisms. Passive DNA demethylation has been found in mitotic cells. During cell division the pattern of DNA methylation is maintained by DNMT1 (Sharif et al., 2007; Berkyurek et al., 2014). If this DNMT is inhibited or its activity is impaired or decreased, the new synthetized DNA strand is not methylated any more, allowing to a gradual decrease of cytosine methylation during the following divisions. Active DNA demethylation occurs both in plants and mammals and requires enzymes and reactions able to remove the methyl group located on the pyrimidine ring of cytosine. A single enzyme capable of breaking the strong Carbon-Carbon bound in order to directly demethylate the cytosine has not been found yet. However, cytosine demethylation can occur through a series of chemical reactions of deamination and/or oxidation.

One possible way involves the base excision repair (BER) pathway. 5mC is deaminated by activation-induced cytidine deaminase/apolipo-protein B mRNA-editing enzyme complex (AID/APOBEC) and converted to a thymine. The base mismatch is subsequently repaired by the BER pathway, through the insertion of an unmethylated cytosine. The nucleotide excision repair (NER) is an alternative method to remove 5mC, which is generally used to repair DNA containing bulky lesions caused by exposure to chemicals or radiation. GADD45a and GADD45b have been implicated in NER dependent DNA demethylation (Barreto et al., 2007; Ma et al., 2009). The most interesting pathway proposed to participate in active demethylation of DNA is the oxidative demethylation. Anjana Rao’s group found ten eleven translocation enzyme (TET) proteins as the mammalian homologs of the trypanosome proteins J-binding protein 1 (JBP1) and JBP2, enzymes able to oxidize the 5-methyl group of thymine. For the first time they demonstrated that TET1, a Fe(II)/α-ketoglutarate-dependent dioxygenase, is able to catalyse the conversion of 5mC to 5-hydroxymethylcytosine (5hmC) *in vitro* (Tahiliani et al., 2009). Later, a similar enzymatic activity was found for TET2 and TET3 too (Ito et al., 2010). Moreover, 5hmC can be further oxidize to 5-formyl-cytosine (5fC) and 5-carboxy-cytosine (5caC; He et al., 2011; Ito et al., 2011), however their levels are significantly lower than that of 5hmC. The discovery of TET enzymes has raised the intriguing possibility for new roles of different oxidised states of cytosine in gene expression control and in the dynamic regulation of DNA methylation.

5hmC is particularly abundant in the central nervous system (CNS) relative to many other tissues. Intriguingly, 5hmC in the brain is 10-fold higher than in embryonic stem cells (ESCs; Globisch et al., 2010; Song et al., 2011) highlighting a possible role for hydroxyl methylation in the epigenetic control of neuronal function.

## Cytosine Covalent Modifications and Neuronal Plasticity

In the last decade, emerging evidence emphasizes epigenetic modifications as key players in brain plasticity. Recently, DNA methylation and hydroxyl methylation have been the major focus, being involved in a variety of fundamental processes in the CNS: neuronal stem cell differentiation (Juliandi et al., 2010), environmental programming of molecular, hormonal and behavioral responses (Meaney and Szyf, 2005; Champagne, 2008; Caldji et al., 2011) and synaptic plasticity (Heyward and Sweatt, 2015).

DNMT1 and DNMT3a levels remain high in post mitotic neurons, implying a specific role for these enzymes in the adult brain that goes beyond the classic view of *de novo* and maintenance DNMT. For instance the double, but not the single, knock-out (KO) mouse model of *Dnmt3a* and *Dnmt1* in forebrain excitatory neurons shows impairment in hippocampal plasticity and learning and memory (Feng et al., 2010), suggesting redundant activity of these DNMT in mature neurons. Furthermore, DNMT1 and DNMT3a have been demonstrated to be the protagonists of a variety of different forms of neuronal plasticity in the adult, such as fear- related memory (Miller and Sweatt, 2007), drug addiction and emotional behavior (LaPlant et al., 2010), and age associate decline in cognitive abilities (Oliveira et al., 2012). Together with the modulation in DNMT expression, changes in CG methylation on the promoter of specific plasticity genes have shaded new light on the role of experience-dependent methylation of DNA in the CNS. Indeed, GADD45b is necessary for activity dependent DNA demethylation on *Bdnf exon IX* and *fibroblast growth factor 1* (*Fgf1*) promoters, critical genes involved in adult neurogenesis in the hippocampus (Ma et al., 2009). Moreover, fear conditioning is associated with rapid methylation and transcriptional silencing of the memory suppressor gene *Pp1* and demethylation and transcriptional activation of the synaptic plasticity gene *Reelin*, indicating dynamic and opposite methyl cytosine changes during memory consolidation in the hippocampus (Miller and Sweatt, 2007).

DNA methylation might be exploited by neurons to achieve dynamic changes in transcriptional activity during memory formation that could be maintained with acquisition of the memory trace. In line with this idea, contextual fear conditioning has been shown to cause hypermethylation of the *calcineurin* gene in the prefrontal cortex of rats and notably, this increase in 5mC persists after 1 month from the training even if *calcineurin* levels go back to their baseline. Nonetheless, rats fail to display normal memory under infusion of DNMT inhibitors in the anterior cingulate, a region of the dorsa-medial prefrontal cortex involved in retrieval (Frankland et al., 2004), 30 days after the training (Miller et al., 2010). These results strongly support the idea that DNA methylation is a critical epigenetic mark for remote memory stability in the adult brain. Importantly, the size of the change in methylation is not big, rarely surpassing a 20% change. Whether this is due to the fact that physiological activity exerts a modulatory effect on methylation levels, or to the inherent difficulty of using neural tissue homogenates that comprises different cell types possibly diluting cell specific effects, is still to be ascertained. Studies dedicated to cell-specific investigations are needed to solve this issue (see below).

Finally, a comprehensive study gave a global overview of the activity-dependent neuronal methylome in the granules of the dentate gyrus, showing how external stimuli can quickly modify the methylation landscape of mature neurons *in vivo* (Guo et al., 2011a). It is worth noting that the level of 5hmC is very high in the brain and that the classic bisulfite sequencing analysis is not able to distinguish between 5mC and 5hmC (Huang et al., 2010). Base-resolution sequencing of 5hmC and 5mC using a method that can distinguish between the two modifications found that 4.2% of total cytosines are 5mC and 0.87% are 5hmC, which represents a 20% difference in quantification if 5hmC are incorrectly called as 5mC (Lister et al., 2013). Not surprisingly, the high content of 5hmC in the brain has caught the attention of neuroscientists in the last few years prompting them to elucidate the influence of hydroxyl methylation on epigenetic dynamics in neurons. The first genome-wide map of 5hmC in neuronal tissue was performed on mouse cerebellum and hippocampus at different stages of mouse life: development, adulthood and ageing. The authors found a strong increase in 5hmC from the early postnatal stage of development to adulthood suggesting a robust correlation with neurodevelopment (Szulwach et al., 2011). It would be interesting and meaningful to extend this type of high throughput analysis to all the different neurons and glial cells present in the diverse brain areas to understand how plastic and cell-specific are the brain methylome and hydroxylmethylome. A first step in this direction was made by Heintz’s group. Using the translating ribosome affinity purification technique to isolate the cell-specific transcriptome, and fluorescence activated cell sorting of EGFP/L10a labeled soma to isolate the cell-specific methylome and hydroxylmethylome, they performed a comparison between the transcriptome and the genome wide distribution of 5mC and 5hmC in cerebellar cell types *in vivo*. Purkinje cells, granule cells, and the terminally differentiated and specialized Bergmann glial cells were compared. Interestingly, specific gene expression and covalent DNA modifications profile were present in the different types of cells (Mellén et al., 2012).

Although many efforts have been made to explain the role of 5hmC, its function as a stable epigenetic mark or a labile reaction intermediate is still an open question that has been addressed in previous reviews (Hahn et al., 2014; Sun et al., 2014). It has been proposed that 5hmC might be the intermediate of a quick demethylation, re-methylation and again oxidation cycle. In line with this hypothesis are the high level of DNMT3a in neuronal nuclei and its association with TDG (Feng et al., 2005; Li et al., 2007). However, this model does not explain why 5fC and 5caC are so low with respect to 5hmC, as detected by sensitive mass spectrometry techniques (Liu et al., 2013). Thus, 5hmC could be a stable mark exerting a specific regulatory role. 5hmC is abundant in gene bodies and promoter regions of active genes providing correlative evidence for a role in gene expression regulation. This role is also suggested by the observation that 5hmC is recognized by unique epigenetic readers in diverse cells (Spruijt et al., 2013). For example, methyl CpG binding protein 2 (MeCP2), one of the most studied 5mC readers, is able to bind hydroxylated cytosine with affinity comparable to methylated cytosine. Moreover, the binding of MeCP2 to 5hmC in expressed genes seems to facilitate transcription through organization of dynamic chromatin domains. However, the functional significance of this interaction remains unclear. Intriguingly, R133C substitution, one the most well known mutations in MeCP2 underlying Rett syndrome (RTT), loses its specific binding capability to 5hmC despite retaining the binding to 5mC (Mellén et al., 2012). This discovery gives new insights on the physiological role of MeCP2 in chromatin structure and organization and might help to better understand crucial molecular mechanisms for the pathophysiology of RTT. RTT is an example of a disease caused by an alteration of a 5mC and 5hmC reader, however several other neurodevelopmental disorders of genetic origin are caused by mutations of factors involved in DNA methylation (see Box 1), underscoring the importance of DNA methylation for brain function.

Box 1 **DNA Methylation and Neurodevelopmental Disorders**

Several neurodevelopmental disorders are characterized by mutations in epigenetic proteins implicated in DNA methylation mechanisms, suggesting that disrupting this chromatin feature can induce severe developmental defects.

1. **Rett Syndrome (RTT)**. RTT is a postnatal neurological disorder that results in serious intellectual and motor disability. In the majority of cases, it is caused by loss of function mutations in the X-linked gene Methyl CpG binding protein 2 (MeCP2; Della Sala and Pizzorusso, 2014). Originally MeCP2 was thought to bind only methylated CG dinucleotide (Lewis et al., 1992), recently it was discovered that it can bind with high affinity 5hmC and mCH (Mellén et al., 2012; Guo et al., 2014).
2. **Immunodeficiency-centromeric instability-facial anomalies (ICF) syndrome**. ICF syndrome is a rare autosomal recessive disorder characterized by immunodeficiency, intestinal dysfunction, mental retardation, psychomotor impairment and particular facial features (Ehrlich, 2003). In 60–70% of ICF patients there is a mutation in the *Dnmt3b* gene (Xu et al., 1999), generally in the catalytic domain. The result is chromosomal instability caused by hypomethylation of pericentromeric chromatin. Moreover bisulfite sequencing analysis in limphoblastoid cells from ICF patients showed a marked decrease in DNA methylation occurring in heterocromatic regions, satellite repeats and transposons, clearly underling a robust remodeling of the epigenetic landscape in consequence of DNMT3b aberrant function (Heyn et al., 2012).
3. **Alpha-thalassemia X-linked intellectual disability (ATRX) syndrome**. Persons affected by ATRX syndrome present distinctive craniofacial features, genital anomalies, severe developmental delays, hypotonia, intellectual disability, and mild-to-moderate anemia secondary to alpha-thalassemia. The disorder is caused by mutations in ATRX gene, a SWI/SNF helicase/ATPase. Intriguingly, ATRX has been demonstrated to interact with MeCP2 and in MeCP2 null-brain ATRX delocalizes from the heterocromatic foci suggesting that this interaction is important for ATRX and MeCP2 function in the context of a correct neuronal development (Nan et al., 2007). Furthermore, mutations in ATRX give rise to modifications in the pattern of DNA methylation of several highly repeated sequences (Gibbons et al., 2000).
4. **2q23.1 Microdeletion syndrome**. This rare neurodevelopmental disorder is characterized by partial or complete deletion of Methyl binding protein 5 (MBD5). The subjects show severe intellectual deficits, epilepsy and autistic features (Talkowski et al., 2011).

In addition, abnormalities in genomic imprinting (exclusive expression of specific genes from only one parent, induced by the silencing of one of the two alleles through DNA methylation and other epigenetic mechanisms) can cause neurodevelopmental disorders such as Prader-Willi syndrome and Angelman syndrome (Weissman et al., 2014). Expansion and aberrant methylation of CGG repeats on the first exon of *Fmr1* gene are responsible for the loss of its protein product FMRP and the development of Fragile X syndrome, a commonly inherited form of intellectual disability and one of the genetic leading causes for autism spectrum disorders (ASD; Wang et al., 2012).

Finally, genome wide association studies have been starting to reveal how changes in DNA methylation might be involved in ASD, a very complex class of neurodevelopmental diseases caused by a combination of genetics and environment (for a comprehensive review about the argument, see Loke et al., 2015).

A fundamental question to address has been if dynamic hydroxylation and methylation of DNA in neurons are activity-dependent. The adult brain has been the most investigated scenery so far. Electroconvulsive stimulation induces demethylation of *Bdnf exon IX* and* Fgf1* promoter in the dentate gyrus through a mechanism involving TET1 dependent hydroxylation and APOBEC1 deamination (Guo et al., 2011b). Similarly, status epilepticus and epileptic tolerance are able to induce a particular methylation profile in the hippocampal genome of adult mice confirming how an aberrant neuronal activity can quickly and specifically modulate the epigenetic landscape in neuronal cells (Miller-Delaney et al., 2012). TET1 is regulated by electrical stimulation in the hippocampus and its hydroxylase activity drives active demethylation. Overexpression of TET1 and of a catalytically inactive form of TET1 in the hippocampus impairs long-term associative memory and expression of plasticity genes, suggesting a new, however not yet investigated, hydroxylase-independent role of TET1 in regulating transcription and memory formation (Kaas et al., 2013). Although the deficiency of TET1 in mouse is compatible with embryonic and postnatal development (Dawlaty et al., 2011), loss of *Tet1* alters the maintenance of the neuronal progenitor pool causing a reduction in neurogenesis in the dentate gyrus. The proposed mechanism consists in the transcriptional repression of neurogenesis-related genes due to hypermethylation of their promoter. Consistent with an impaired adult neurogenesis, *Tet1* null mice displayed defective cognitive functions, specifically in the acquisition of spatial memory (Zhang et al., 2013). Furthermore, in *Tet1* null mouse model long term depression (LTD) is abnormally enhanced and they fail to exhibit memory extinction, a form of inhibitory learning that provides a basis for an adaptive control of cognition. Cognitive and synaptic deficits are accompanied by gene expression impairments, in particular by a downregulation in neuronal activity regulated genes such as *Npas4*, whose promoter is hypermethylated both in the cortex and hippocampus of *Tet1* ko animals (Rudenko et al., 2013). Recently, TET1 has been implicated in the molecular and behavioral effects of cocaine. After chronic cocaine administration TET1 is downregulated in the nucleus accumbens of adult mice and 5hmC is increased at putative enhancer regions and gene bodies (Feng et al., 2015). These results are not totally in agreement with previous research on learning and memory, in which a decrease in TET1 caused less 5mC hydroxylation and thus to hypermethylayion and transcriptional repression (Kaas et al., 2013; Rudenko et al., 2013; Zhang et al., 2013). However, the addiction data focuses on changes in 5hmC in gene bodies more than on promoters, and on a genome wide scale, suggesting a much more complicated level of epigenetic regulation for hydroxylated DNA and TET enzymes in the brain.

Although TET1 is the less expressed isoform in the adult brain (Szwagierczak et al., 2010), it has been the protagonist of the majority of the research in the field. Recent studies have concentrated their attention on the role of TET3, the most abundant TET in both cortex and hippocampus, in behavioral adaptation and homeostatic plasticity (Li et al., 2014; Yu et al., 2015). TET3 is activity-dependent in cortical neurons *in vitro* and is induced after fear extinction learning in the infra-limbic prefrontal cortex of rodents. Furthermore, extinction is able to cause a TET3 dependent redistribution and accumulation of 5hmC in the prefrontal cortex, creating a transcriptional permissive chromatin environment especially for a key gene in the organization of the postsynaptic side of the inhibitory synapse, such as *Gephyrin* (Li et al., 2014). In cultured hippocampal neurons TET3 levels are regulated by electrical activity and TET3 itself is involved in modulation of basal excitatory synaptic transmission and synaptic scaling through changes in the glutamate receptor Glur1 surface expression (Yu et al., 2015). Finally, TET2 has been identified as the TET enzymes responsible for 5hmC mark in the fetal brain of putative regulatory regions that are demethylated and activated in the adult animal, suggesting that TET2 mediates 5mC demethylation during neuronal development (Lister et al., 2013). However, there are no reports implicating TET2 in activity-dependent gene expression control. Unlike DNMT enzymes that seem to have, at least in part, a redundant role in the adult brain (Feng et al., 2010), each TET enzyme seems to play a more specific role. Future research will unveil the specific function and regulation of TET activity in brain plasticity.

It is becoming clear that covalent modifications of DNA are a powerful epigenetic tool to fine-tune neuronal functions and synaptic plasticity. Hydroxylation and methylation of cytosine have been extensively studied in the context of embryonic development and in the adult brain, giving exciting insights on their role in neuronal differentiation and reprogramming of pluripotent stem cells or in complex behavioral processes, such as learning and memory. In the next paragraph, we will review recent data showing that experience-dependent regulation of DNA methylation and hydroxyl methylation could be even more dynamic in juvenile animals during developmental critical periods of heightened plasticity (Tognini et al., 2015).

## Experience-Dependent DNA Methylation During Post-Natal Development

The visual system has long been one of the most investigated models to study experience-dependent plasticity because visual experience can be easily manipulated and the subsequent effects can be measured at the anatomical, physiological and molecular level. The maturation of the visual circuitry starts before the eye-opening and therefore before the onset of vision, although experience is necessary for a correct development of the visual system, especially during a specific window of time called the critical period. In rodents, the critical or sensitive period covers an interval between 3 and 7 weeks of life. During this sensitive period of heightened plasticity, experience is particularly effective in producing permanent and extensive modifications of cortical organizations (Spolidoro et al., 2009; Espinosa and Stryker, 2012; Levelt and Hübener, 2012; Takesian and Hensch, 2013).

**Figure 1:**
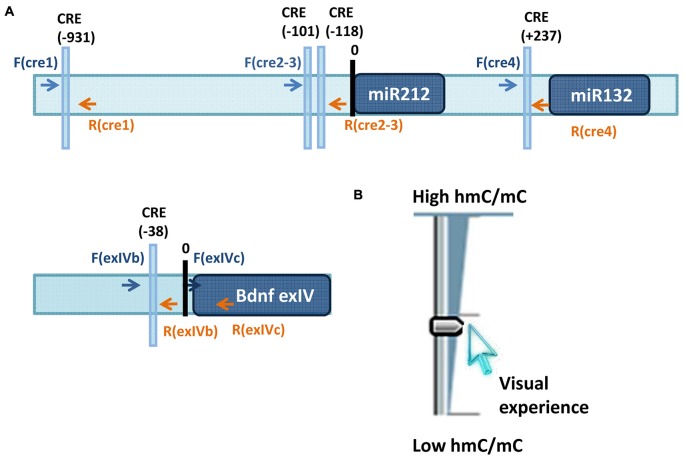
**(A)** Loci studied in Tognini et al. (2015) to assess visual experience regulation of 5mC and 5hmC. Arrows point the location of the primers used for immunoprecipitation analysis of 5mC and 5hmC. **(B)** Sketch illustrating the results obtained on *miR-212/132* CRE1 and CRE2–3 promoters and on promoter region of *BDNF exon4*. Visual experience modulated in opposite direction 5mC and 5hmC abundance at these loci resulting in an increase of 5hmC/5mC ratio.

Ocular dominance plasticity (ODP) consists in a rapid modification in the responses of visual cortical neurons, which results from unbalanced visual input from the two eyes. A large variety of mechanisms have been proposed to contribute to ODP and critical period plasticity in general (Levelt and Hübener, 2012). For example, extracellular matrix composition and axon myelinisation (Pizzorusso et al., 2002, 2006; McGee et al., 2005), maturation of inhibitory circuits that changes the excitatory/inhibitory balance making the circuitry less favorable to plasticity (Hensch and Fagiolini, 2005; Gandhi et al., 2008; Yazaki-Sugiyama et al., 2009; Iurilli et al., 2013; Saiepour et al., 2015), CREB dependent gene expression (Pham et al., 1999; Mower et al., 2002; Cancedda et al., 2003) and histone post-translational modifications (Putignano et al., 2007) have all been proposed to contribute to critical period closure. Recently, a CREB dependent microRNA, miR-132 has been shown to be a key player in the regulation of ODP in the developing visual cortex (Mellios et al., 2011; Tognini et al., 2011). MiR-132 expression levels correlate with changes in epigenetic marks, such as acetylation and phosphorylation of the histone H3, induced by visual stimuli manipulations (Tognini et al., 2011). Monocular deprivation (MD, the suture of one eye), the classic paradigm to study ODP, is able to downregulate miR-132 in the visual cortex contralateral to the deprived eye. Strikingly, counteracting miR-132 downregulation through the infusion of chemically modified miRNA mimic in the cortex, or further reducing miR-132 through injection of a lentivirus expressing a miR132-sequestering sponge, during MD completely prevents ODP in young mice (Mellios et al., 2011; Tognini et al., 2011). Interestingly, the miR-132 genomic locus, that contains both miR-132 and miR-212 transcripts (Tognini and Pizzorusso, 2012), lies in a region enriched in CG dinucleotides, although the significance of miR-132 locus CpG methylation in neural cells has been totally unexplored. In a new study, Tognini et al. (2015) demonstrated that the effects of visual deprivation on miR-132 expression is regulated by a dynamic switch in DNA methylation and hydroxyl methylation after 3 days of MD in the visual cortex of juvenile mice. Moreover, *Gadd45a, b* and *g* are all downregulated. GADD45 family members participate in active DNA demethylation and have been previously shown to be involved in plasticity (Leach et al., 2012; Sultan et al., 2012; Sultan and Sweatt, 2013). Their reduction in the deprived cortex, combined with the increase in DNMT expression, results in an enhancement of DNA methylation in the deprived cortex on specific DNA loci. Single-base resolution analysis of 5mC on the promoter region of well-known plasticity genes downregulated by MD during the critical period, miR-132 and *Bdnf exon4* transcript (Hong et al., 2008; Mellios et al., 2011; Tognini et al., 2011), demonstrates a significant increase in DNA methylation in the visual cortex contralateral to the deprived eye. Remarkably, 5hmC shows an opposite behavior on the same DNA loci suggesting that sensory inputs are able to dynamically modulate cytosine states to control experience-dependent gene expression (Figure 1). It is worth noting that MD does not modify DNA methylation and hydroxyl methylation patterns in adult mice strongly connecting these dynamic changes to cortical plasticity levels. Strikingly, the pharmacological inhibition of DNMT counteracts experience-dependent downregulation of miR-132 and *Bdnf exon 4* in the deprived visual cortex. To analyze the importance of DNMT activity for experience-dependent regulation of gene transcription in the visual cortex at whole transcriptome level, the authors performed RNA sequencing analysis of deprived mice and of mice treated with the DNMT inhibitor RG108. It was found that for about 13.5% of the genes regulated by MD, DNMT inhibition prevents the effects of visual deprivation. Since DNMT inhibition never exerts the opposite effect, i.e., enhancing the effect of deprivation, these data suggest that DNMT activity is necessary for a significant component of the transcriptional program activated by MD during the critical period. The DNMT-dependent part of the transcriptional program activated by MD is essential for visual cortical plasticity, indeed electrophysiological investigation revealed that ODP is completely blocked in the cortex treated with DNMT inhibitor. Taken together, these results reveal that visual deprivation has opposite effects on DNA methylation and hydroxyl methylation on specific genomic regions and suggest that these modifications could be brought about by the visual regulation of DNMT, GADD45 and APOBEC-3 expression and possibly their DNA binding. For the first time, dynamic modifications of DNA have been implicated in mediating visual experience-dependent modulation of transcription of a selected gene set necessary for the molecular processes underlying ODP.

This finding represents just the first step to unravel how sensory inputs can modulate covalent cytosine modifications in order to refine the cortical circuits during development. The study points out how visual experience through DNA methylation and hydroxyl methylation dynamics is able to influence the physiological property of cortical neurons during the critical period. It would be interesting to check if these DNA methylation changes represent a long lasting epigenetic memory trace capable of affecting future behavioral responses in adulthood.

## Non-CpG DNA Methylation

Until few years ago, cytosine methylation was thought to be restricted to CpG sites, because of their intrinsic symmetry that facilitates the transmission of this epigenetic mark through cell divisions (Schübeler, 2015). However, CpG are not the only sites undergoing methylation. First reported in embryonic and pluripotent cells (Ziller et al., 2011; Lister et al., 2013), particularly high level of methylation in non-CpG cytosines (mCH, where H stands for adenine A, thymine T or cytosine C) has been found in the adult mouse and human brain genome (Xie et al., 2012; Varley et al., 2013) and recently, in almost all human tissues (Schultz et al., 2015). Both the non-CpG methylated loci and the target sites found in the adult brain are different from those in ESCs: CAC is the preferred trinucleotide in neuronal cells and CAG in ESCs (Lister et al., 2013; Guo et al., 2014). After these first observations, many questions have been coming up about the distribution, function and cell-specificity pattern of mCH in the adult brain. Using genome wide single-base resolution analysis it has been found that mCH is absent in the fetal cortex and accumulates in the mouse and human brain in the early postnatal life becoming the predominant form of DNA methylation (~53% mCH vs. ~47% mCG; Lister et al., 2013). Remarkably, the analysis of sorted neuronal and glial cells revealed that high levels of mCH are strongly enriched in neurons and just sparse in glia suggesting that this form of DNA methylation might be more relevant for neuronal-specific functions. Moreover, gene ontology analysis shows that genes hypermethylated in CH in glial cells are implicated in neuronal and synaptic development and functions. On the other hand, the same genes are hypomethylated in CH and CG in neurons, indicating that mCH might have a transcriptional repressive role for neuronal-specific genes in the glial genome (Lister et al., 2013). Notably, the increase in mCH coincides with a postnatal developmental window characterized by a burst in synaptogenesis followed by activity-dependent pruning of excess synapses meaning that neurons could use this modification to sculpt their gene expression during critical periods. The identification of the functional role of mCH is an emerging research field that is still under intense study (for review, see He and Ecker, 2015). However, recent anti-correlation results between mCH levels and gene expression suggest that mCH has the same repressive function of mCG in differentiated cells (Lister et al., 2013; Guo et al., 2014; Schultz et al., 2015). *In vitro* studies manipulating mCH content to assess its role on transcriptional activity using mCH methylated plasmids expressing GFP as a reporter gene (Guo et al., 2014) confirmed the hypothesis that mCH is repressive for gene transcription. However, it is worth noting that mCH repressive action cannot be generalized to all cell types. For instance, a correlation between high levels of mCH and high levels of expression has been observed in ESCs (Chen et al., 2011). These differences are possibly due to distinct types of DNA methylation writers and readers present in each cell type. ESCs have a high DNMT3b/DNMT3a expression ratio as opposed to mature neurons showing very low levels of DNMT3b (Okano et al., 1999; Lister et al., 2013; Tognini et al., 2015). DNMT3b is able to catalyze the CH methylation reaction and recent work suggests that is likely to be the major enzyme for mCH in human ESCs (Liao et al., 2015). DNMT3b selectively binds to the bodies of transcribed genes possibly by interacting with H3K36Me3, a histone mark typical of actively transcribed genes in ESCs (Baubec et al., 2015). This mechanism exemplifies how mCH could have opposite functional effects in different cell types.

As already mentioned, CpG methylation is catalyzed by DNMT. DNMT3a shows a developmental expression timing overlapping with the postnatal brain accumulation of mCH (Lister et al., 2013). The role of DNMT3a in CH methylation is also supported by data showing that knockdown with adeno-associated virus of DNMT3a produces a reduction of mCH levels that is not obtained with DNMT1 silencing (Guo et al., 2014). Furthermore, ChIP results demonstrate the direct binding of DNMT3a to mCH enriched regions (Lister et al., 2013). Therefore, DNMT3a seems to be the principal writer of mCH in differentiated neurons. Strikingly, MeCP2, a classic reader of mCG, can bind also to mCH (Guo et al., 2014; Chen et al., 2015; Gabel et al., 2015); in particular mCA is its preferential target with comparable or even more affinity than mCG. mCA is enriched in gene bodies of long genes (>100 kb), preferentially expressed in the CNS (Gabel et al., 2015). Thus, the deleterious effects of MeCP2 loss of function in RTT could be due to the upregulation of a specific set of genes consisting in the genes with length >100 kb. Importantly, the transcription of long genes is dependent on topoisomerases (King et al., 2013) raising the possibility that pharmacological inhibition of topoisomerases might be beneficial in RTT. In agreement with this theory, topoisomerase inhibition in knocked-down MeCP2 neuronal cultures induces a dose-dependent reversal of long gene expression. It remains to demonstrate if the *in vivo* use of this pharmacological compound could reverse also the phenotype of RTT mouse models. Considering the importance of DNA methylation for neuronal functions, it is likely that future studies will discover a role for mCH also in other brain pathologies.

## Conclusions and Perspectives

Epigenetic regulation of gene expression is one of the most fascinating research areas. Many exciting discoveries have been made during the last decade, however we still need to address many questions. Scientists have started to unravel the existence of an intricate “neuronal epigenetic code”, important for cells differentiation, brain development and plasticity both in physiology and pathology. The brain has an exceptional and unique epigenetic feature with respect to all the other tissues in our body, both referring to the abundance of specific epigenetic marks: 5-hydroxyl methylation on cytosine, non-CG methylation, and to an extremely plastic epigenetic landscape due to the continuous stimulation from the environment. We are just beginning to understand how specific epigenetic marks are regulating gene expression in response to exogenous and endogenous inputs and how remodeling of the chromatin milieu can contribute to the modifications in the structure and anatomy of neuronal circuitry.

Another level of complexity to the study of chromatin architecture in neurons consists in the regulation of enzymes responsible to read, write and erase epigenetic marks. It has been demonstrated that cofactors (i.e., NAD) and metabolites (i.e., Acetyl- CoA, Beta-hydroxybutirate, alpha-ketoglutarate) are able to influence the activity of enzymes, such as acetyl transferases, deacetylases and TET (Xu et al., 2011; Shimazu et al., 2013; Pietrocola et al., 2015). Therefore, the nutritional state and the lifestyle can modulate the activity of epigenetic enzymes and subsequently the chromatin structure, connecting again environment and gene expression. Although still in its infancy, the epigenetic link between brain plasticity and metabolic state seems to be a new promising and exciting research field.

Finally, the mammalian brain is a very complicated and heterogeneous organ characterized by functionally different areas and a huge variety of cell populations. Neural and glial cells are characterized by a unique microenvironment, a broad ranging interconnectivity and indeed, might have a different and exclusive epigenome and transcriptome. So far, DNA methylation and histone posttranslational modifications related to plasticity processes have been assessed mostly in tissue homogenates, comprising together heterogeneous cells (excitatory and inhibitory neurons, glial cells etc.) and potentially masking the diversity of cell-specific epigenetic marks or gene expression. The solution to this challenge is a single-cell analysis (i.e., laser capture microdissection, fluorescence activated cell sorting) that present some obstacles due to high costs, low yield of specific cell types, and cell manipulation prior to analysis. An effort in this direction has been recently made via the utilization of INTACT (isolation of nuclei tagged in specific cell types (Deal and Henikoff, 2010) to isolate and examine the genetic and epigenetic features of some classes of neocortical neurons in the mouse brain: excitatory pyramidal neurons, parvalbumin-expressing and vasoactive intestinal peptide-expressing interneurons (Mo et al., 2015). This study underlined fundamental differences between the transcriptome and the methylome in different neuronal subtypes in the healthy brain. It would be interesting to apply this technique to the developmental brain and to neurological disease models to unravel how plastic is the neuron-specific genome and epigenome in response to environmental and pathological cues.

The future advancement in technology will hopefully shed new light on the intricate epigenetic mechanisms regulating neuronal plasticity and function providing novel directions to create better therapeutic interventions for neuropsychiatric symptoms in humans.

## Funding

The project on epigenetics of the Pizzorusso lab is supported by Telethon, Epigenomics Flagship Project EPIGEN MIUR-CNR and AIR (Associazione Italiana sindrome di Rett).

## Conflict of Interest Statement

The authors declare that the research was conducted in the absence of any commercial or financial relationships that could be construed as a potential conflict of interest.
